# Semaphorin 3A Contributes to Secondary Blood–Brain Barrier Damage After Traumatic Brain Injury

**DOI:** 10.3389/fncel.2019.00117

**Published:** 2019-03-26

**Authors:** Mengchen Yang, Xiaoxue Wang, Yueshan Fan, Yaqing Chen, Dongdong Sun, Xin Xu, Jianhao Wang, Gang Gu, Ruilong Peng, Tianyu Shen, Xilei Liu, Fanjian Li, Yi Wang, Dong Wang, Hongtao Rong, Zhenying Han, Xiangliang Gao, Qifeng Li, Keyuan Fan, Yuhua Yuan, Jianning Zhang

**Affiliations:** ^1^Department of Neurosurgery, Tianjin Medical University General Hospital, Tianjin, China; ^2^Key Laboratory of Injuries, Variations and Regeneration of Nervous System, Tianjin Neurological Institute, Tianjin, China; ^3^Tianjin Medical University, Tianjin, China; ^4^Department of Clinical Laboratory Diagnostics, Tianjin Medical University General Hospital, Tianjin, China; ^5^The Second Hospital of Tianjin Medical University, Tianjin, China; ^6^Tianjin Chest Hospital, Tianjin, China

**Keywords:** traumatic brain injury, blood–brain barrier, Semaphorin 3A, miRNA-30b-5p, oxygen-glucose deprivation

## Abstract

Semaphorin 3A (SEMA3A) is a member of the Semaphorins family, a class of membrane-associated protein that participates in the construction of nerve networks. SEMA3A has been reported to affect vascular permeability previously, but its influence in traumatic brain injury (TBI) is still unknown. To investigate the effects of SEMA3A, we used a mouse TBI model with a controlled cortical impact (CCI) device and a blood–brain barrier (BBB) injury model *in vitro* with oxygen-glucose deprivation (OGD). We tested post-TBI changes in SEMA3A, and its related receptors (Nrp-1 and plexin-A1) expression and distribution through western blotting and double-immunofluorescence staining, respectively. Neurological outcomes were evaluated by modified neurological severity scores (mNSSs) and beam-walking test. We examined BBB damage through Evans Blue dye extravasation, brain water content, and western blotting for VE-cadherin and p-VE-cadherin *in vivo*, and we examined the endothelial cell barrier through hopping probe ion conductance microscopy (HPICM), transwell leakage, and western blotting for VE-cadherin and p-VE-cadherin *in vitro*. Changes in miR-30b-5p were assessed by RT-PCR. Finally, the neuroprotective function of miR-30b-5p is measured by brain water content, mNSSs and beam-walking test. SEMA3A expression varied following TBI and peaked on the third day which expressed approximate fourfold increase compared with sham group, with the protein concentrated at the lesion boundary. SEMA3A contributed to neurological function deficits and secondary BBB damage *in vivo*. Our results demonstrated that SEMA3A level following OGD injury almost doubled than control group, and the negative effects of OGD injury can be improved by blocking SEMA3A expression. Furthermore, the expression of miR-30b-5p decreased approximate 40% at the third day and 60% at the seventh day post-CCI. OGD injury also exhibited an effect to approximately decrease 50% of miR-30b-5p expression. Additionally, the expression of SEMA3A post-TBI is regulated by miR-30b-5p, and miR-30b-5p could improve neurological outcomes post-TBI efficiently. Our results demonstrate that SEMA3A is a significant factor in secondary BBB damage after TBI and can be abolished by miR-30b-5p, which represents a potential therapeutic target.

## Introduction

Traumatic brain injury (TBI) is a high-incidence disease that can harm human health, and with the development of society, the morbidity of this disease has tended to increase (Cope et al., 2012). The mortality of TBI is 10.8%, and the disability rate is 12%. TBI has become the fifth major cause of death among people under 40 years old, and young adults account for approximate 70% of all TBI patients (Sen, 2017).

Due to the comprehensive effect of primary injury and secondary injury, TBI may cause various types of damage in the clinical setting (Blennow et al., 2012; Khalili et al., 2017; Sen, 2017). The primary injury of TBI leads to a series of effects including neural damage, intracerebral hemorrhage and primary blood–brain barrier (BBB) disruption, which result in subsequent pathological events in the central nervous system (CNS) (Blennow et al., 2012; Jiang et al., 2017). BBB consists of various cells (endothelial cell, astrocytes, pericytes, and neurons), and acts as a dynamic interface between circulation and CNS (Alluri et al., 2018). The function of BBB in regulating the exchange of substances protects the CNS from damage caused by pathogenic microorganisms or other macromolecular substances in the circulation (Eliceiri et al., 1999; Marmarou, 2007). Secondary brain damage resulting from the interaction of primary pathological events and products is characterized by secondary BBB damage which occurs from hours to days following TBI (Marmarou, 2007; Ge et al., 2015). The secondary destruction of BBB by reason of the primary pathological events and products results in the further damage including brain edema, intracranial hypertension, inflammation and even poor neurological prognosis (Zweckberger et al., 2006; Ge et al., 2015; Xiong et al., 2017). As a result, improving secondary BBB damage is a significant approach for treatment and improving prognosis after TBI. Currently, clinical therapy focuses mainly on decompressive craniectomy, the evacuation of intracranial hematoma, and dehydrant treatment, which aims to treat the symptoms of brain edema with limited therapeutic effects. The effect of hypothermia therapy on TBI patients is also contradicted by the National Institutes of Health (NIH) (Mena et al., 2011). In conclusion, an efficient therapeutic method that addresses the cause of secondary BBB damage is urgently needed.

Semaphorin 3A (SEMA3A) is a member of a membrane-associated and secreted proteins family with more than 20 types identified (Yang et al., 2018), which participate in the construction of nerve networks. SEMA3A has long been known in nervous system as an axonal guidance factor to guide these growing axons by repelling them or preventing them from entering certain regions (Yazdani and Terman, 2006). A wide range of studies on SEMA3A also have been performed in different physiological and pathological processes, including cardiogenesis, angiogenesis, vasculogenesis, tumor metastasis, osteoclastogenesis and immune regulation (Takamatsu et al., 2010). Duh (2011) and Joyal et al. (2011) have reported that SEMA3A prevents angiogenesis in the retina and destructs the blood–eye barrier. The antiangiogenic effect is associated with the integrity of endothelial cells and SEMA3A alters the integrity of these cells through VE-cadherin serine phosphorylation and internalization (Le Guelte et al., 2012). The integrity of BBB depends on inter-endothelial-cell junction and its complex structures (Sayeed et al., 2018). The complex structures of inter-endothelial-cell junction which include microvascular endothelium, tight junctions and adherens junctions, can help to regulate BBB permeability and functional activities (Bazzoni et al., 2000; Zlokovic, 2008). However, whether SEMA3A participates in TBI and how SEMA3A carries out its biological functions remain unknown.

Based on the database analysis of TargetScan, Miranda and miRDB, we found that miRNA-30 was closely related to SEMA3A. Previous research indicated that miR-30c was related to SEMA3A in neurogenesis (Sun et al., 2016) and MiR-30b promotes axon outgrowth of retinal ganglion cells by inhibiting SEMA3A expression (Han et al., 2015). Furthermore, miR-30a-5p was also reported to significantly suppress the inflammation responses following spinal cord injury (Fu et al., 2018). In circulation system and urinary system, miR-30 family was also demonstrated the positive effect following ischemia injury (Shen et al., 2015; Gu et al., 2016). However, which factors can regulate the secretion of SEMA3A in brain tissue following TBI needs further investigation.

We investigated the alterations in SEMA3A after TBI to determine whether SEMA3A can act as a factor in secondary BBB damage and regulate neurological function post-TBI. Furthermore, our results indicated OGD injury can induce the secretion of SEMA3A *in vitro* through imitating hypoxic pathological status. However, miR-30b-5p was confirmed to regulate SEMA3A expression efficiently and abolished the effect of SEMA3A on the integrity of the BBB *in vivo* and *in vitro*.

## Materials and Methods

### Animals

Adult male C57BL/6 mice weighing 20–25 g were purchased from the Beijing Vital River Laboratory Animal Technology Co., Ltd. All mice were maintained in the animal facilities of Tianjin Medical University General Hospital with free access to food and water in a temperature-controlled (20 ± 2°C) and humidity-controlled (55 ± 5%) vivarium under a 12 h light/dark cycle. All animal experiments were approved by the Ethics Committee of Tianjin Medical University (Tianjin, China). Furthermore, all the experimental protocols for this study were performed in accordance with the NIH Guide for the Care and Use of Laboratory Animals and approved by the Tianjin Medical University Animal Care and Use Committee.

### Experimental Design

Five experimental procedures were performed to investigate the influence of SEMA3A on secondary BBB damage post-TBI and its regulation factor.

In experiment 1, the post-TBI changes in the expression level of SEMA3A and its related receptors were determined by western blotting. The mice used for this experiment were divided into five groups: sham group, TBI 1Day group, TBI 3Day group, TBI 7Day group, and TBI 14Day group, with each group consisting of six mice. The concentrated area was determined by Double-Immunofluorescence Staining and the mice were assigned to two groups of six: sham group and TBI group (third Day post-TBI).

In experiment 2, we studied the effect of SEMA3A on neurological deficits and BBB damage post-TBI. The TBI mice were divided into five groups, each comprising six mice: the Sham group; the PBS group, in which PBS (2 μl) was injected into the lateral ventricle; the siRNA-control group, which was transfected with siRNA-control; the SEMA3A group, in which the SEMA3A protein (200 ng/μl) was injected into the lateral ventricle; and the siRNA group, which was transfected with siRNA-SEMA3A. mNSSs and modified beam-walking test were used to evaluate the neurological function and tested at 1st, 3rd, 7th, and 14th days post-CCI. EB dye extravasation assay, brain water content and BBB tight junction protein western blotting test were used to evaluate BBB damage post-TBI and tested at third day following TBI (SEMA3A group served as the positive control and the administered dose was 200 ng/μl).

In experiment 3, the OGD injury’s effect on the expression of SEMA3A was studied with western blotting *in vitro*, and bEnd.3 cells were divided into sham group and OGD group. The influence of SEMA3A on endothelial cell barrier was evaluated by FITC-Dextran transport studies, HPICM and BBB tight junction protein western blotting test. In FITC-Dextran transport studies and BBB tight junction protein western blotting test, bEnd.3 cells were divided into Control group, OGD+PBS group, OGD+siRNA-C group, OGD+siRNA-SEMA3A group and OGD+SEMA3A group. In HPICM test, bEnd.3 cells were divided into control group, SEMA3A group, OGD group, OGD+siRNA-C group, OGD+siRNA-SEMA3A group (OGD+SEMA3A group and SEMA3A group served as positive control, and the administered dose was 200 ng/μl).

In experiment 4, we used RT-PCR to study the expression level change of miR-30b-5p post-TBI *in vivo* and *in vitro*. The mice were divided into Sham 1D group, Sham 3D group, Sham 7D group, Sham 14D group, TBI 1D group, TBI 3D group, TBI 7D group, and TBI 14D group, with each group consisting of six mice. Then, the association between SEMA3A and miR-30b-5p was measured by western blotting and Luciferase reporter assay.

In experiment 5, we used brain water content, mNSSs and modified beam-walking test to measure the neuroprotective effect of miR-30b-5p during TBI. The mice were divided into the Sham group, TBI+PBS group, TBI+agomir-C group and TBI+agomir group. Furthermore, we also detected whether miR-30b-5p protected brain following TBI by regulating SEMA3A. The mice were divided into TBI+agomir-C+SEMA3A group and TBI+agomir+SEMA3A group. SEMA3A was injected on second day post-TBI with the dose of 200 ng/μl.

### Controlled Cortical Impact (CCI) Model

The TBI model was established in C57BL/6 mice by a CCI device (eCCI-6.3 device, Custom Design and Fabrication, United States). The mice adapted to the environment for 1 week before the experiments. The mice were anesthetized with 10% chloral hydrate (3 mg/kg) via an intraperitoneal injection before surgery. The mice were then placed in a stereotaxic frame. After the surgical site was clipped and cleaned, to expose the dura, a 4.0-mm diameter hole was drilled in the right parietal bone (2.0 mm lateral to the sagittal sutures, 2.0 mm posterior to the bregma). To induce moderate TBI, the CCI device was set to a depth of 2 mm, a velocity of 4.5 m/s and a dwell time of 200 ms (Ge et al., 2018; Xu et al., 2018). Immediately after the injury, the skull was closed by sterilized medical bone wax and the incision was sutured with 6-0 silk sutures. The mice were placed on a heating pad to recover from anesthesia and moving normally (the waking time post-CCI is 0.5–1 h). And then, the mice were removed back to the animal facilities of Tianjin Medical University General Hospital and set in independent cages. After that, the mice were monitored the incision (redness, swelling, discharge), appetite, fecal, urine production and specific signs related to the CCI every day. The mice in the sham group were subjected to all procedures except CCI. All the animal experimental protocols for this study were performed in accordance with the NIH Guide for the Care and Use of Laboratory Animals and approved by the Tianjin Medical University Animal Care and Use Committee.

### Cell Culture of the Mouse Brain Endothelial Cell Line (bEnd.3) and Oxygen-Glucose Deprivation (OGD) Cell Injury Model

The bEnd.3 cells were purchased from the BeNa Culture Collection (Suzhou, China) and cultured in DMEM-basic medium (Gibco, United States) with 10% FBS and 1% penicillin/streptomycin (Thermo Fisher Scientific). The cells were maintained in a culture chamber (Thermo Fisher Scientific) at 37°C with 5% CO_2_.

To imitate BBB injury *in vitro*, OGD was carried out. The culture medium was replaced with glucose-free endothelial cell medium (ECM), and the cells were placed into an anaerobic chamber (HERACELL 150i, Thermo Fisher Scientific) for 4 h at 37°C with the CO_2_ level at 5% and the O_2_ level at 1%. The cell viability was evaluated by Cell Counting Kit-8 (Dojindo, Shanghai, China) and maintained above 65%.

### Western Blot Analysis

The mice were anesthetized with 10% chloral hydrate (3 mg/kg) via an intraperitoneal injection and sacrificed through transcardiac perfusion to eliminate test error caused by proteins expressed by blood cells. The brains were removed from the mice and homogenized in radioimmunoprecipitation assay (RIPA) buffer (Beyotime) mixed with phenylmethylsulfonyl fluoride (PMSF) (1 mM) for 30 min. The homogenates were then centrifuged for 10 min (12,000 rpm, 4°C), and the supernatants were collected. The supernatants were mixed with 4X loading buffer and boiled at 95°C for 10 min. The total protein content of the protein samples was determined through a bicinchoninic acid (BCA) protein assay kit (Thermo), and the proteins were separated by sodium dodecyl sulfate/polyacrylamide gel electrophoresis (SDS/PAGE). Next, the proteins were transferred to a polyvinylidene fluoride (PVDF) membrane (Roche, Canada) and blocked with 5% non-fat dry milk in Tris-buffered saline (TBS) for 2 h at room temperature. The blots were then incubated with primary antibodies overnight at 4°C and with the appropriate horseradish peroxidase (HRP)-conjugated secondary IgG for 1 h at room temperature. Finally, the blots were developed with an enhanced chemiluminescence (ECL) system (Millipore, Billerica, MA, United States), and the expression levels of protein samples were quantified with ImageJ software. β-Actin served as a loading control (Sun et al., 2017). More information about the antibodies is presented in Table 1.

**Table 1:** Antibodies for Western blot.

Antibody	Calalog#	Vendor	Dilution	Molecular weight (kDa)
Semaphorin 3A	ab23393	Abcam	1:250	95
Neuropilin-1	AF566-SP	NOVUS	1:1000	150
Plexin A1	AF4309-SP	NOVUS	1:1000	200
VE-Cadherin	ab205336	Abcam	1:1000	90
VE-Cadherin (phospho Tyr731)	YP0808	Immunoway	1:1000	130
β-Actin	3700	CST	1:1000	45
Peroxidase-conjugated anti-Rabbit IgG (H + L)	ZB-2305	ZSGB-BIO	1:5000	
Peroxidase-conjugated anti-Goat IgG (H + L)	ZB-2306	ZSGB-BIO	1:5000	

### Double-Immunofluorescence Staining

The mice were grouped into a TBI group and a sham group, which were anesthetized with 10% chloral hydrate (3 mg/kg) through an intraperitoneal injection and sacrificed through transcardiac perfusion [cold phosphate-buffered saline (PBS) and 4% paraformaldehyde] 72 h after TBI. The brains were removed and embedded in optimal cutting temperature (OCT) medium (Sakura, Oakland, CA, United States). The brains were subsequently sliced into 8-mm-thick coronal sections. After blocking with 3% bovine serum albumin (BSA) for 30 min at 37°C, the sections were incubated with the appropriate primary antibody mixture, which was mixed with an anti-SEMA3A antibody (1:75, Abcam, ab23393) and an anti-CD31 antibody (1:100, Abcam, Ab24590), overnight at 4°C. The samples were then incubated with Alexa Fluor-conjugated anti-rabbit IgG (1:500, Molecular Probes) for 1 h at room temperature, and the nuclei were counterstained with 4’,6-diamidino-2-phenylindole (DAPI) for 5 min.

### siRNA Transfection

siRNA transfection was carried out *in vivo* with the method reported previously (Chen et al., 2009). siRNA-SEMA3A (0.5 nmol, RiboBio, Guangzhou, China) and siRNA-control (0.5 nmol, RiboBio, Guangzhou, China) were diluted with the same volume of Entranster TM -*in vivo* transfection reagent (Engreen, Beijing, China). The mixed solutions were injected intracerebroventricularly (i.c.v.) into the lateral ventricle by using stereotactic coordinates (1.5 mm posterior to the bregma, 1.0 mm right lateral to the sagittal suture, and 2 mm in depth). The CCI treatment was administered immediately post-transfection.

siRNA transfection was carried out *in vitro* through Lipofectamine-3000 (Thermo Fisher Scientific). siRNA-SEMA3A (1 nmol, RiboBio Biotechnology, Guangzhou, China) and siRNA-control (1 nmol, RIBOBIO, Guangzhou, China) were diluted with the same volume of Lipofectamine-3000 and added to the culture medium for 5 h. The OGD treatment was performed at 36 h post-transfection.

### Modified Neurological Severity Scores (mNSS)

mNSSs were carried out as previously reported (Chen et al., 2001) to evaluate neurological function. The observer was blinded to the experimental conditions and treatments. All the mice were tested at 1, 3, 7, and 14 days post-CCI. Lower scores indicated better neurological function. If the mice died before 14 days post-CCI, their data was not included in the statistical analysis.

### Motor Function Testing

Motor function was evaluated through a modified beam-walking task, as previously reported (Feeney et al., 1982). Before TBI induction, we trained the mice to walk along a narrow beam and enter a darkened box at the end of the beam using stimulation with bright light and loud noise. As the mouse entered the goal box, all the stimulations were stopped immediately. The tests were carried out on the 1st, 3rd, 7th, and 14th days post-TBI and recorded by an observer who was blinded to the experimental conditions and treatments. Shorter times indicated better motor function.

### Evans Blue (EB) Dye Extravasation Assay

The mice were injected with a 2% EB solution (Sigma-Aldrich, 5 ml/kg) through the femoral vein on the third day after CCI, as previously reported (Tchantchou and Zhang, 2013; Xu et al., 2018). After 2 h, the mice were transcardially perfused with PBS and sacrificed. The lesioned hemispheres were dissected, weighed and incubated in *N*,*N*-dimethylformamide (1 ml/100 mg) at room temperature for 48 h. Then, the mixtures were centrifuged at 1,000 rpm for 5 min. The supernatants were collected, and optical density (OD) values were determined by a spectrophotometer (λ = 632 nm). The quantity of extravasated EB dye was measured according to the standard curve.

### Brain Water Content

Brain water content was evaluated through the wet–dry weight method on the third day post-CCI, as previously reported (Yan et al., 2011; Sun et al., 2017; Xu et al., 2018). Briefly, the mice were sacrificed, and the brains were obtained without transcardiac perfusion. The lesioned hemispheres were isolated and weighed directly. Then, the lesioned hemispheres were dried in an electrothermostatic blast oven at 80°C for 72 h. Brain water content (%) was calculated using the following formula: (wet brain weigh-dry brain weight)/wet brain weight × 100%.

### FITC-Dextran Transport Studies

The bEnd.3 cells were cultured in transwells (Corning, 0.4 μm) and divided into a negative control group, an OGD+PBS group, an OGD+siRNA-C group, an OGD+siRNA-SEMA3A group and an OGD+SEMA3A group, which served as a positive control. After the different groups had been treated separately, we added FITC-dextran (Sigma-Aldrich, 4 kDa, 2 mg/ml) into the medium in the apical chamber and collected the medium in the basolateral chamber after 1 h. Then, we tested the OD value of the collected medium with a spectrophotometer.

### Hopping Probe Ion Conductance Microscopy (HPICM) Scanning

The HPICM system consisted of an ICnano scanner controller (Ionscope) and a sample scan head SH01 (Ionscope, Melbourn, Cambridgeshire, United Kingdom), which was placed on an inverted TiU microscope (Nikon, Tokyo, Japan). Cell movement in the horizontal X–Y–Z direction was measured by a 25-μm LISA piezo stage (P-753.21C, Physik Instrumente) and two 100-μm PIHera piezo stages (P-621.2C, Physik Instrumente, Karlsruhe, Germany), which were managed by the ICnano controller. The ion current between the nanopipette tip and the cell surface was monitored by an external Axon Multi Clamp700B amplifier (Molecular Devices, Sunnyvale, CA, United States). The nanopipettes, which were filled with Leibovitz’s L15 medium (Thermo Fisher Scientific), were pulled from the borosilicate glass (OD 1.00 mm, I.D. 0.59 mm, VitalSense Scientific Instruments, Wuhan, China) with a laser-based puller (Model P-2000, Sutter Instruments, Novato, CA, United States) (Chen et al., 2013). First, we continuously scanned the cells, to which we added PBS or the SEMA3A protein during the scanning procedure, for 2 h. The OGD group, OGD+siRNA-C group and OGD+siRNA-SEMA3A group were scanned after they had been treated separately. All the primary data was analyzed by SICM Image Viewer software (Ionscope).

### Quantitative Real-Time PCR

RNA was harvested from the acquired tissues on the 1st, 3rd, 7th, and 14th days post-TBI and from the cultured cells post-OGD with TRIzol reagent (Invitrogen). The RNA was reverse transcribed into single-stranded complementary DNA with the PrimeScript RT Reagent Kit (TaKaRa). The single-stranded complementary DNA was amplified with SYBR Premix Ex Taq II (TaKaRa) and calculated by an MJ Research real-time PCR system (Bio-Rad, Hercules, CA, United States). The reaction was carried out under the following conditions: 95°C for 2 min, followed by 45 cycles of 95°C for 15 s and 62°C for 1 min. All the primers are presented in Table 2. U6 was used as an endogenous control.

**Table 2:** Polymerase chain reaction primer sets in real-time PCR.

	Primer sequence, 5′–3′	
Gene	Forward	Reverse
*miR-30b-5p*	GCGCGTGTAAACATCCTACAC	AGTGCAGGGTCCGAGGTATT
*U6*	CTCGCTTCGGCAGCACA	AACGCTTCACGAATTTGCGT

### Transfection of miR-30b-5p *in vivo* and *in vitro*

To downregulate or upregulate the expression level of miR-30b-5p in mice, the mice were randomly divided into the TBI+PBS group, the TBI+antagomir control group, the TBI+antagomir group, the TBI+agomir control group, and the TBI+agomir group. The oligomers were transfected at a dose of 800 ng per mouse by the Entranster TM-*in vivo* transfection reagent (Engreen, Beijing, China). The mixed solutions were injected i.c.v. into the lateral ventricle by using stereotactic coordinates (1.5 mm posterior to the bregma, 1.0 mm right lateral to the sagittal suture, and 2 mm in depth). The CCI treatment was administered 24 h post-transfection.

In *in vitro* experiments, the culture cells were randomly divided into the OGD+PBS group the OGD+inhibitor control group, the OGD+inhibitor group, the OGD+mimic control group, and the OGD+mimic group. The solutions were transfected into the cells separately by Lipofectamine-3000 (Thermo Fisher Scientific) at a dose of 100 nM. The OGD treatment was performed at 36 h post-transfection.

To evaluate the transfection efficacy of miR-30b-5p, real-time PCR was carried out to detect changes in the expression levels of miR-30b-5p in mouse brain tissue and bEnd.3 cells. Oligomers, mimic, inhibitor and control reagent were all purchased from RiboBio Biotechnology.

### Luciferase Reporter Assay

To determine whether miR-30b-5p directly targeted the *Sema3a* gene, a luciferase reporter assay was performed. Luciferase reporter constructs were created by ligating *Sema3a* 3′ untranslated region (UTR) fragments containing the predicted binding sites. The pGL3- *Sema3a* -3′UTR construct was then inserted into the pGL3 control vector containing the SV40 promoter (Promega, Madison, WI, United States) using the XbaI enzyme. In addition, a mutant (Mut) luciferase reporter was generated from the wild-type (WT) luciferase reporter by deleting the binding site for miR-30b-5p.

For the reporter assay, 293T cells were cultured in 24-well plates. The WT or Mut *Sema3a*-3′UTR was co-transfected with miR-21-3p agomir or the agomir negative control (sequences listed in Table 2; GenePharma) into 293T cells with Lipofectamine-3000. The cells were harvested, and luciferase activity was evaluated with a dual-luciferase reporter system (Promega, Madison, WI, United States) after 48 h of incubation.

### Statistical Analysis

All data were based on the group sizes of *n* = 6 and every sample was tested three times to confirm. Data of the mNSS test and beam-walking test were analyzed using repeated measures ANOVA followed by LSD *post hoc* analysis. For other data, statistical comparisons were analyzed using one-way ANOVA followed by LSD *post hoc* analysis or Student’s *t*-test. All data were expressed as the mean ± SEM and normally distributed which were analyzed by SPSS statistical software (version 22.0, IBM). A *p*-value less than 0.05 was considered significant.

## Results

### SEMA3A Expression Levels After TBI

**FIGURE 1:**
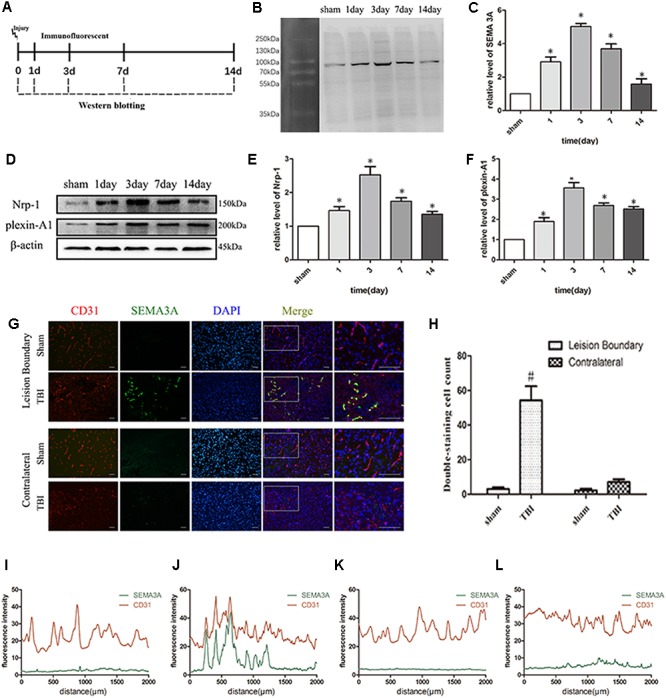
TBI altered the expression of SEMA3A and its related receptors, Nrp-1 and plexin-A1. **(A)** Schematic diagram of the experimental design. **(B,C)** Quantitative data from western blotting illustrating the time course of SEMA3A following TBI, with an increase on the first day post-TBI and peaked on the third day. **(D–F)** Quantitative data from western blotting illustrating the time course of the related receptors of SEMA3A following TBI, with an increase on the first day post-TBI and peaked on the third day. **(G–L)** Double-immunostaining data indicating that SEMA3A was mainly secreted and concentrated at the lesion boundary after TBI. **(I)** Fluorescence intensity of lesion boundary of sham group. **(J)** Fluorescence intensity of lesion boundary of TBI group. **(K)** Fluorescence intensity of contralateral of sham group. **(L)** Fluorescence intensity of contralateral of TBI group. Scale bar = 50 μm. The data are expressed as the mean ± SEM, and *n* = 6 for each group. ^∗^*p* < 0.05 vs. sham groups, ^#^*p* < 0.01 vs. sham group and TBI contralateral group.

To determine if SEMA3A is associated with TBI, biochemical assays were performed in the above groups from 1st day to 14th day after TBI (Figure 1A). We found that the expression of SEMA3A and related receptors, Plexin-A1 and Nrp-1, were dramatically elevated on the first day after TBI and peaked on the third day (Figure 1B–F). Double-immunofluorescence staining for SEMA3A and endothelial cells was used to investigate the difference in expression levels between the lesion boundary and the contralateral tissue, which revealed a concentrated expression of SEMA3A at the lesion boundary after TBI (Figure 1G–L). These data may demonstrate that SEMA3A is associated with a destructive process or a reparative process after TBI.

### SEMA3A Contributes to the Neurological Deficits Induced by TBI

**FIGURE 2:**
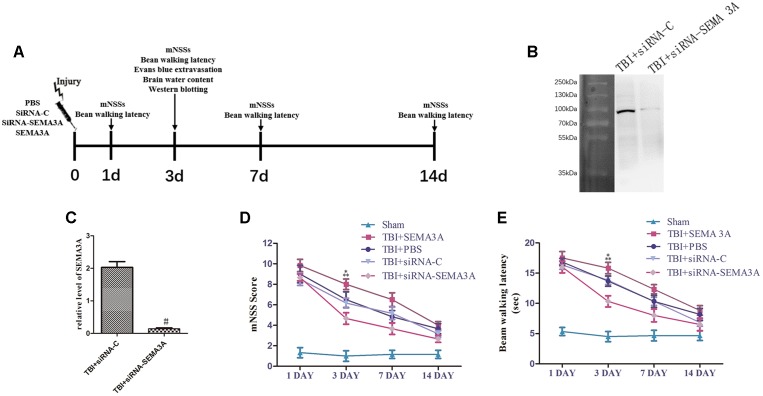
Influence of SEMA3A on neurological outcomes following TBI. **(A)** Schematic diagram of the experimental design. **(B,C)** The expression of SEMA3A was blocked by siRNA-SEMA3A *in vivo*. **(D,E)** We used mNSSs to test neurological function **(D)** and beam balance tests **(E)** to evaluate motor function on the 1st, 3rd, 7th, and 14th days post-CCI. The downregulation of SEMA3A significantly decreased mNSS scores and reduced the times on the beam balance test on the third day after injury. The data are expressed as the mean ± SEM, and *n* = 6 for each group. ^#^*p* < 0.01 vs. control groups, ^∗^*p* < 0.05 TBI+siRNA-SEMA groups vs. siRNA-control groups, ^∗∗^*p* < 0.05 TBI+SEMA3A groups vs. TBI+PBS groups.

To evaluate the effect of SEMA3A on neurological deficits after TBI, we compared the neurological function of TBI mice with mNSS scores and beam-walking test from the 1st day to the 14th day post-TBI (Figure 2A) and decrease SEMA3A expression with siRNA (Figure 2B,C).

In the mNSS test (Figure 2D), there was no significant difference between the PBS group and the siRNA-control group, which exhibited a decreasing trend from the 1st day to the 14th day post-TBI, illustrating that all mice sustained relatively comparable injuries. The SEMA3A group, which served as the positive control group, was compared with the PBS group on the third day post-TBI, which showed that SEMA3A could effectively aggravate neurological deficits. In addition, in a comparison of the siRNA-control group and the siRNA group on the third day post-TBI, neurological function began to recover, with an obvious decrease in mNSSs. All these tendencies were echoed in the beam-walking test (Figure 2E), which indicated that SEMA3A was a significant factor that participated in the process associated with the neurological deficits induced by TBI.

### SEMA3A Contributes to BBB Leakage *in vivo*

**FIGURE 3:**
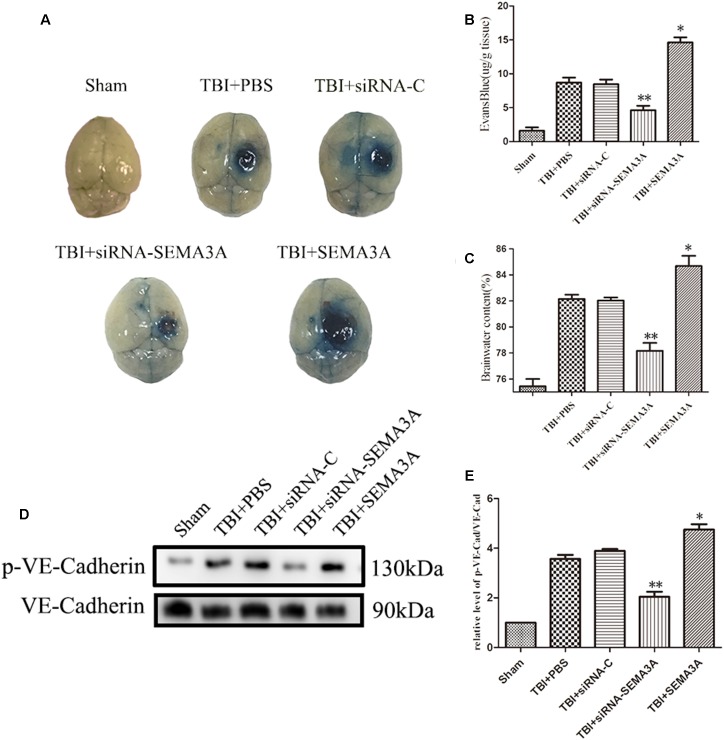
The impact of SEMA3A on BBB leakage after TBI. **(A)** The general view of the EB dye extravasation assay in brains of injured mice on the third day after injury. **(B)** Quantitative analysis of the EB dye extravasation assay presented in **(A). (C)** The brain water content was measured on the third day after injury. SEMA3A can significantly increase BBB leakage and edema, and all these negative effects post-TBI can be efficiently decreased by blocking the expression of SEMA3A. **(D,E)** The p-VE-cadherin/VE-cadherin ratio was examined on the third day after injury through western blotting, and the results illustrated that the downregulation of SEMA3A can decrease VE-cadherin serine phosphorylation post-TBI. The data are expressed as the mean ± SEM, and *n* = 6 for each group. ^∗^*p* < 0.05 TBI+SEMA3A groups vs. TBI+PBS groups, ^∗∗^*p* < 0.05 TBI+siRNA-SEMA groups vs. siRNA-control groups.

We investigated whether SEMA3A can carry out the same function in the context of BBB leakage after TBI with Evans Blue (EB) dye extravasation assay and brain water content measurement at 72 h post-injury according to the previous research (Li et al., 2011; Sun et al., 2017; Xu et al., 2018). The results demonstrated that SEMA3A can increase the EB leakage from blood vessels into the brain tissue; however, this expression was effectively blocked when SEMA3A was blocked by siRNA on the third day after TBI (Figure 3A,B). Following the appearance of this discrepancy, brain edema was obviously decreased due to the decline in SEMA3A levels (Figure 3C). It was previously reported that the serine phosphorylation and internalization of the adherens junction molecule VE-cadherin upon SEMA3A stimulation play key roles in controlling endothelial barrier function. We aimed to detect variations in brain endothelial junctions after TBI. We found that the siRNA group had a decreased p-VE-cadherin/VE-cadherin ratio (Figure 3D,E), which indicated that SEMA3A may act as a factor that can alter the barrier function of brain endothelial junctions and contribute to the destruction of the BBB post-TBI.

### OGD Induces Changes of SEMA3A Expression in Endothelial Cells and Contributes to BBB Leakage Induced by OGD

**FIGURE 4:**
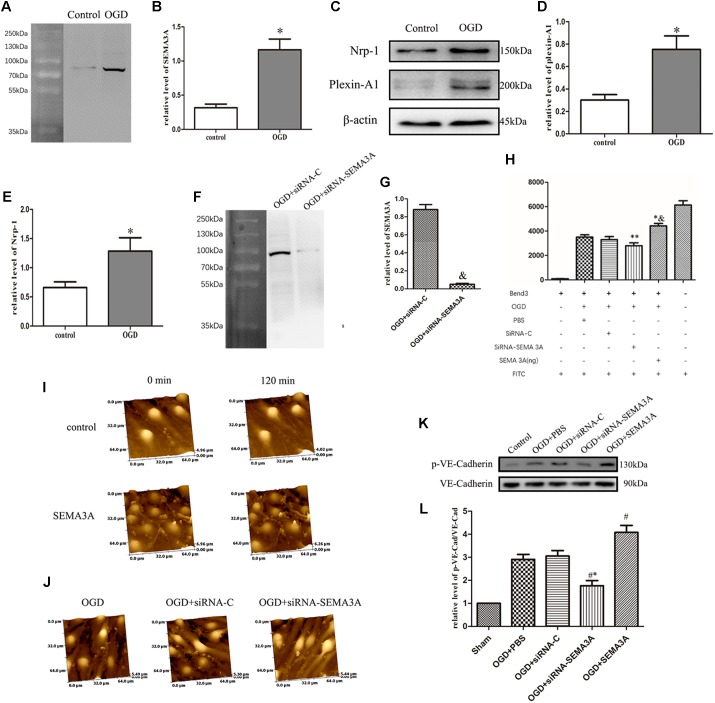
OGD induced changes in SEMA3A expression in endothelial cells and contributes to BBB leakage induced by OGD. **(A–E)** To investigate the causes of the alterations of SEMA3A post-injury, we treated endothelial cells (bEnd.3) with OGD for 4 h and tested protein expression levels with western blotting. The results indicated that compared with those in the control group, which was cultured under normoxic conditions, the expression levels of SEMA3A **(A,B)**, Nrp-1 and plexin-A1 **(C–E)** in the OGD group significantly increased. **(F,G)** The expression of SEMA3A was blocked by siRNA-SEMA3A *in vitro*. **(H)** FITC-dextran transport studies showed that the amount of FITC-dextran that was transported through the transwell into the lower chamber dramatically increased in the OGD group and the SEMA3A group. This amount decreased when SEMA3A was blocked. **(I)** We treated bEnd.3 cells with the SEMA3A protein and observed the morphological changes by HPICM. The results illustrated that SEMA3A can efficiently increase defects of the BBB. **(J)** Observations of the integrity of the barrier by HPICM indicated that the destruction of the barrier that is induced by OGD is efficiently impaired by the downregulation of SEMA3A expression. **(K,L)** Testing of tight junction changes post-OGD with western blotting revealed that SEMA3A contributes to VE-cadherin serine phosphorylation. The data are expressed as the mean ± SEM, and *n* = 6 for each group. ^∗^*p* < 0.01 vs. control group, ^&^*p* < 0.01 vs. control group, ^∗∗^*p* < 0.05 vs. control groups, ^∗&^*p* < 0.05 vs. OGD+PBS group, ^#∗^*p* < 0.01 vs. siRNA-control group, ^#^*p* < 0.05 vs. OGD+PBS group.

Oxygen-glucose deprivation is the most common way to imitate hypoxic injury *in vitro* (Beckmann et al., 2018), and Hypoxic injury generally occurs after TBI (Salehi et al., 2017). Furthermore, OGD is also a recognized method to establish BBB injury model *in vitro* (Ge et al., 2018). To investigate the changes in SEMA3A expression after trauma injury *in vitro*, we treated the endothelial cells (bEnd.3) with OGD and then tested the expression levels of SEMA3A, plexin-A1 and Nrp-1. OGD could efficiently increase the expression levels of SEMA3A and its related receptors (Figure 4A–E), which indicated that SEMA3A is associated with a destructive process after OGD injury *in vitro*.

Regarding the previous results, we predicted that SEMA3A may act as a significant factor in BBB injury. To determine the function of SEMA3A in BBB leakage induced by OGD, we established a BBB model with endothelial cells (bEnd.3) *in vitro* and evaluated BBB leakage with FITC-dextran transport studies and HPICM. We found that SEMA3A can efficiently open the BBB model and increase leakage. From morphological observations through HPICM, we found that SEMA3A led to the formation of cavities in the BBB model *in vitro*. However, the leakage induced by OGD can be efficiently decreased by blocking the expression of SEMA3A (Figure 4F–J). Next, we examined the expression of p-VE-cadherin and VE-cadherin and calculated the p-VE-cadherin/VE-cadherin ratio. The results revealed that blocking the expression of SEMA3A can decrease VE-cadherin serine phosphorylation in bEnd.3 cells post-OGD (Figure 4K,L) and demonstrated that SEMA3A affected BBB integrity after OGD by changing the function of tight junctions.

### miR-30b-5p Regulates SEMA3A Expression Following TBI *in vivo* and *in vitro*

**FIGURE 5:**
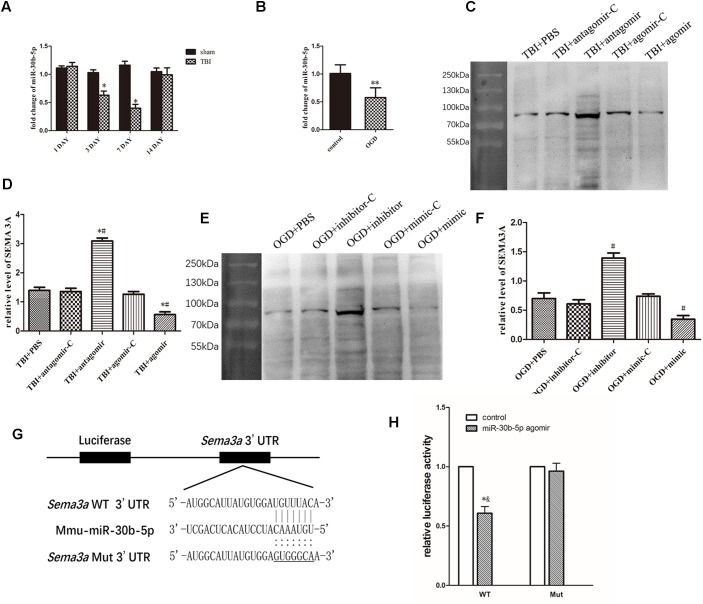
miR-30b-5p regulated SEMA3A expression following injury *in vivo* and *in vitro*. **(A)** Compared with those of the sham groups, the RT-PCR results indicated that the expression level of miR-30b-5p in CCI mice decreased and reached its lowest value on the seventh day post-injury. **(B)** In an *in vitro* experiment, the expression level of miR-30b-5p decreased in bEnd.3 cells post-OGD. **(C,D)** In the CCI mouse model, the downregulation of miR-30b-5p increased the expression level of SEMA3A, and the upregulation of miR-30b-5p decreased SEMA3A levels. **(E,F)** These results were repeated in the OGD model in bEnd.3 cells. **(G)** Schematic representation of the potential binding sites for miR-30b-5p in the *Sema3a* 3′UTR. Seed sequences of the WT (*Sema3a* WT 3′UTR) and mutant (*Sema3a* Mut 3′UTR) luciferase reporters are shown in the binding site. **(H)** Data from the luciferase assay indicated that miR-30b-5p inhibited the luciferase activity of the WT but not the Mut 3′UTR reporter construct, suggesting that miR-30b-5p could directly target *Sema3a* and downregulate its expression by binding to the 3′UTR sites. The data are expressed as the mean ± SEM, and *n* = 6 for each group. ^∗^*p* < 0.05 vs. sham group, ^∗∗^*p* < 0.05 vs. control group, ^∗#^*p* < 0.01 vs. control groups, ^#^*p* < 0.01 vs. control groups, and ^∗&^*p* < 0.05 vs. control groups.

To study whether miR-30b-5p could regulate SEMA3A expression in central nerve system, we first carried out RT-PCR to assess the miRNA level of miR-30b-5p in brain tissue on the 1st, 3rd, 7th, and 14th days after TBI and in bEnd.3 cells at 4 h post-OGD. We found that the miRNA level of miR-30b-5p in brain tissue decreased after TBI and reached its lowest value on the seventh day (Figure 5A). In addition, the miRNA level of miR-30b-5p in bEnd.3 cells also decreased following OGD (Figure 5B). We then downregulated and upregulated the level of miR-30b-5p *in vivo* and *in vitro*. We found that the level of SEMA3A changed in response to variations in the miR-30b-5p level. miR-30b-5p helped to reduce SEMA3A expression post-injury (Figure 5C–F). Furthermore, to study the sites in the SEMA3A 3′UTR to which miR-30b-5p binds, we used miRDB to find the potential binding site and conducted a luciferase reporter assay. The results showed that miR-30b-5p inhibited the luciferase activity of the WT construct but not the Mut 3′UTR reporter construct (Figure 5G,H), indicating that miR-30b-5p directly targets SEMA3A and downregulates its expression by binding to sites in the 3′UTR of *Sema3a*.

### The Neuroprotective Effect of miR-30b-5p in Mice Following TBI

**FIGURE 6:**
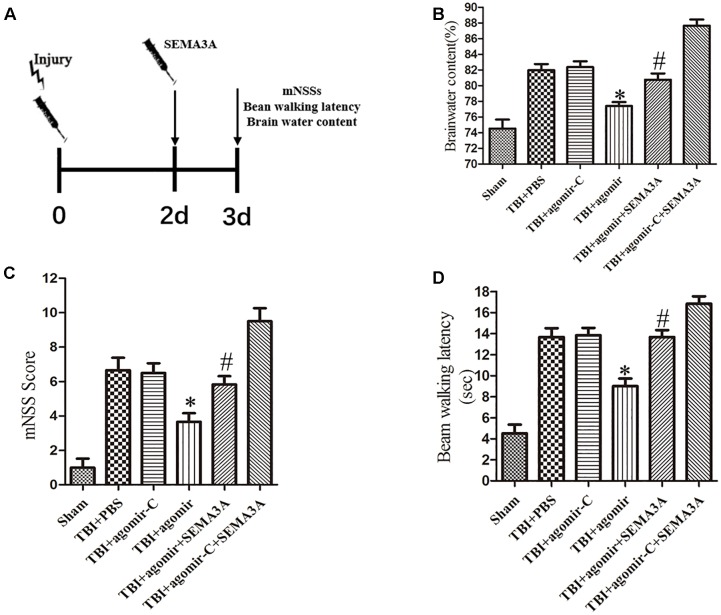
The neuroprotective effect of miR-30b-5p in mice following TBI by regulating SEMA3A. **(A)** Schematic diagram of the experimental design. **(B–D)** The brain water content **(B)**, mNSS score **(C)** and beam balance test **(D)** were measured on the third day after injury. Upregulation of miR-30b-5p could efficiently decrease brain edema, and improve neurological outcomes post-TBI. The negative effect of recombinant SEMA3A protein also can be improved by upregulation of miR-30b-5p. The data are expressed as the mean ± SEM, and *n* = 6 for each group, ^∗^*p* < 0.05 vs. control groups, ^#^*p* < 0.05 vs. TBI+agomir-C+SEMA3A group.

To investigate whether miR-30b-5p could improve neurological outcomes after TBI by regulating SEMA3A (Figure 6A), we carried out brain water content, mNSSs and beam-walking test on the third day post-TBI *in vivo*. Firstly, compared with control groups, miR-30b-5p agomir group could alleviate brain edema following TBI (Figure 6B). Furthermore, according to the results of mNSSs (Figure 6C) and beam-walking test (Figure 6D), we found that the neurological deficits post-TBI could be efficiently improved. Besides, we compared the TBI+agomir+SEMA3A group and TBI+agomir-C+SEMA3A group. SEMA3A recombinant protein were injected on the second day post TBI. The results indicated that upregulation of miR-30b-5p could attenuate the negative effect of SEMA3A on neurological outcomes (Figure 6B–D). All these results echoed the negative influence of SEMA3A, and indicated that miR-30b-5p could be a potential therapy after TBI by inhibiting SEMA3A expression.

## Discussion

The effect of SEMA3A on vascular permeability has been proven in a previous report (Acevedo et al., 2008), but neurological research on SEMA3A is very limited, mainly focusing on ischemia stroke, multiple sclerosis lesions, and particularly the functions of SEMA3A in suppressing axon formation and promoting dendrite growth (Shelly et al., 2011; Costa et al., 2015; Hou et al., 2015; Gutierrez-Franco et al., 2016). However, nothing is known about the effects of SEMA3A during TBI and how it is regulated. Thus, we investigated the effects of SEMA3A on secondary BBB injury after TBI in this study. We found that the expression level of SEMA3A significantly changed in a mouse TBI model. SEMA3A induced by TBI could affect neurologic outcomes. OGD injury can induce the secretion of SEMA3A through imitating hypoxic pathological status. Furthermore, we uncovered convincing evidence that SEMA3A expression can be regulated by miR-30b-5p and the neurological deficits post-TBI could be efficiently improved through upregulation of miR-30b-5p in brain tissue.

Blood–brain barrier is formed by the endothelial cells lining the brain microvessels, under the inductive influence of neighboring cell types including astrocytes and pericytes. The endothelium forms the major interface between the blood and the CNS, and by a combination of low passive permeability and presence of specific transport systems, enzymes and receptors regulates molecular and cellular traffic across the barrier layer (Abbott, 2013). Most of pathological changes on CNS are resulting from the damage of BBB. SEMA3A is widely known as a neuronal guidance factor in CNS, which can participate in the construction of nerve networks by regulating not only growth cone motility but also dendritic development and maturation via Nrp-1/Plexin-A1 receptor complex (Campbell et al., 2001), whereby Plexin-A1 acts as the signal transducer (Acevedo et al., 2008).

To investigate the potential effect of SEMA3A on secondary BBB damage after TBI, we employed a CCI mouse model and observed an increasing trend for the expression levels of SEMA3A, Nrp-1 and plexin-A1 after TBI, and peaked on the third day post-injury. Moreover, Nrp-1 is necessary for vessel development, since Nrp-1 knockouts could impair angiogenesis and cardiovascular development (Takashima et al., 2002; Gu et al., 2003). Together with plexins (plexin-A1) and neuropilins (Nrp-1 and Nrp-2), semaphorins (including SEMA3A) have the ability to interrupt vascular formation and tumor vascularization (Gaur et al., 2009). According to our results, we have sufficient reason to predict that SEMA3A may be responsible for secondary BBB damage post-TBI. BBB disruption is considered to be one of the most significant factors associated with pathological changes post-TBI (Wood, 2018) and has been widely demonstrated to be a transient process that can lead to poor lifelong outcomes (Alluri et al., 2015). We confirmed that SEMA3A contributes to the neurological deficits and BBB leakage post-TBI. All of these negative effects following TBI could be improved by blocking SEMA3A expression. Furthermore, the serine phosphorylation of VE-cadherin due to SEMA3A efficiently led to the dysfunction of tight junctions (Le Guelte et al., 2012). TBI always leads to alterations of VE-cadherin (Hue et al., 2014), and the dysfunction of tight junctions contributes to damage to the integrity of the BBB (Goncalves et al., 2012). In our experiments, we confirmed that SEMA3A could increase VE-cadherin serine phosphorylation post-TBI and weaken cell–cell adhesion, contributing to BBB leakage as a vascular permeability factor after TBI. Brain edema is one of the most serious pathological changes that leads to mortality and disability. Vasogenic brain edema is caused by the destruction of BBB integrity (Blixt et al., 2015). We found that the increase in brain water content post-TBI can be effectively decreased by downregulating the expression of SEMA3A.

Oxygen-glucose deprivation is currently the most frequently used *in vitro* model to imitate BBB injury *in vitro* (Ge et al., 2018) through hypoxic pathological damage (Salvador et al., 2015). Hypoxia is significantly associated with negative effects following TBI and contributes to morbidity and mortality (Chesnut et al., 1993). Some compounds induced by hypoxemia, such as extracellular glutamate, can increase damage following primary damage (Bullock et al., 1998). Endothelial cells (bEnd.3) account for the characteristics of the BBB and are regarded as a suitable model for the BBB *in vitro* (Forster et al., 2005). We first demonstrated that OGD injury triggers the release of SEMA3A and its related receptors (Nrp-1 and plexin-A1). Furthermore, the disruption of BBB integrity and BBB leakage induced by OGD can be significantly repaired by blocking the expression of SEMA3A. From morphological observations through HPICM, we found that SEMA3A led to the formation of cavities in the BBB model *in vitro*. The leakage of the BBB model induced by SEMA3A probably resulted from these cavities. Decreasing the expression level of SEMA3A in bEnd.3 cells can efficiently reduce leakage post-OGD, and fewer cavities can be observed in HPICM. The enhancement of the VE-cadherin function promotes endothelial protection and helps to overcome the defects that result from OGD stress in brain endothelial cells (Ishiguro et al., 2011). The VE-cadherin serine phosphorylation following injury was also confirmed to have reduced by SiRNA-SEMA3A.

miRNAs play crucial roles in physiological and pathological processes post-TBI. Redell et al. (2009) first reported the expression changes and pleiotropy of miRNAs in the pathophysiological processes following TBI. Our group also reported a microarray analysis of miRNA expression in the rat’s cerebral cortex post-TBI (Lei et al., 2009). In previous studies, we demonstrated that the neurological outcomes following TBI could be improved through targeting miRNA-21 and miRNA-124 (Ge et al., 2015, 2016, 2018; Huang et al., 2018). In the previous study, several miRNAs were reported to regulate SEMA3A expression. Rezaeepoor et al. (2018) confirmed that SEMA3A serve as an immune modulator is suppressed by miR-145-5p. Furthermore, Liu et al. (2019) also reported that miR-145-5p suppresses osteogenic differentiation of adipose-derived stem cells by targeting SEMA3A. miR-362 can downregulate the expression of SEMA3A in non-small-cell lung carcinoma (NSCLC) and promote lung cancer metastasis (Luo et al., 2018). In the study of hepatocellular carcinoma, SEMA3A can serve as a target regulated by miR-192-5p to promote the proliferation and metastasis of hepatocellular carcinoma cell (Yan-Chun et al., 2017). The research on microRNA regulating SEMA3A was also pretty extensive in optic nervous system (Baudet et al., 2011; Han et al., 2015; Villain et al., 2018). According to the analysis of TargetScan, Miranda and miRDB, we predicted that miRNA-30 is closely related to SEMA3A. miR-30 has been widely discovered, and it has been proven to act as tumor suppressor in the development of various cancers (Cheng et al., 2012; Zhong et al., 2014; Singh et al., 2017; Zhang et al., 2017). Some studies have also confirmed that miR-30 family inhibited tumor cell growth by changing the tumor cell autophagy. For example, Zhang et al. (2017) found that miR-30d could inhibit autophagy of colon cancer cells and promote cell apoptosis through targeting PI3K and ATG5 (Singh et al., 2017). Furthermore, miRNA-30 has also been reported to change its expression in Alzheimer’s progression (Liu et al., 2014). However, in a recent study, miRNA-30c was confirmed to regulate neurogenesis resulting from SEMA3A in the subventricular zones (Sun et al., 2016). Similarly, miR-30b-5p, a mature miRNA derived from pre-miR-30b, caught our attention. miR-30b was confirmed to contribute to proliferation, migration, and invasion in glioblastoma (Li et al., 2018). Whether miR-30b-5p could also be a regulation factor in CNS, particularly following TBI is still unknown. Based on the results exhibited above, we demonstrated a decreasing trend in the miR-30b-5p expression level, which reached its lowest value on the seventh day following injury in the CCI mouse injury model and the OGD-treated cell injury model. Moreover, we also found that the upregulation or downregulation of miR-30b-5p can decrease or increase the expression of SEMA3A post-injury *in vivo* and *in vitro*, which demonstrated that SEMA3A could be abolished by miR-30b-5p in brain tissue. miR-30b-5p inhibited the luciferase activity of the WT, but not the Mut 3′UTR reporter construct, also suggesting that miR-30b-5p could directly target SEMA3A and downregulate its expression by binding to the 3′UTR sites. In addition, upregulation of miR-30b-5p level in the injured brain by intracerebroventricular infusion of miR-30b-5p agomir acted a better neurological outcome after TBI through regulating SEMA3A. The negative effect of SEMA3A on neurological outcomes could be attenuated by upregulation of miR-30b-5p which indicated that miR-30b-5p could be the potential therapeutic treatment for regulating SEMA3A following TBI.

However, this study had some limitations. In this study, we mainly focused on the effect of SEMA3A on secondary BBB damage following TBI and its regulation factors. In addition, destruction of the BBB always leads to the invasion of hazardous substances into the brain tissue. Several pathological events post-TBI are resulting from the damage of BBB. For our future study, downstream signaling of SEMA3A will be studied to clarify its mechanism in other types of pathological damage induced by TBI. We are studying the mechanism of SEMA3A and miR-30b-5p in regulating BBB damage from two aspects: apoptosis and inflammation. Even though the results of this study were not influenced, the effective time point of knockdown group and recombinant SEMA3A protein group following TBI also need to be considered and regulated to make these them comparable in the further mechanism research.

In summary, the major discovery of this study is that the expression level of SEMA3A increased and miR-30b-5p decreased following TBI induced by CCI treatment in mice and OGD treatment in bEnd.3 cells. The downregulation of SEMA3A levels could alleviate BBB damage post-TBI *in vivo* and *in vitro*, and improve the neurological outcomes of CCI mice. Furthermore, the expression level of miR-30b-5p was altered following injury, and miR-30b-5p could regulate SEMA3A expression post-TBI *in vivo* and *in vitro*. miRNA-30b-5p has also been confirmed to improve neurological outcomes following TBI through regulating SEMA3A. Therefore, SEMA3A is a promising therapeutic target for TBI, and miR-30b-5p could be a potential treatment for the downregulation of SEMA3A.

## Author Contributions

JZ and YY designed the experiments. MY, XW, and YF carried out the experiments, analyzed the experimental results, wrote the manuscript. YC, DS, XX, JW, GG, RP, XL, FL, TS, YW, DW, HR, ZH, XG, QL, and KF took part in the experiments and proposed some suggestions.

## Conflict of Interest Statement

The authors declare that the research was conducted in the absence of any commercial or financial relationships that could be construed as a potential conflict of interest.
